# Aging promotes pro-fibrotic matrix production and increases fibrocyte recruitment during acute lung injury

**DOI:** 10.4236/abb.2014.51004

**Published:** 2014-01

**Authors:** Viranuj Sueblinvong, Wendy A. Neveu, David C. Neujahr, Stephen T. Mills, Mauricio Rojas, Jesse Roman, David M. Guidot

**Affiliations:** 1 Division of Pulmonary, Allergy and Critical Care Medicine, Emory University School of Medicine, Atlanta, GA, USA; 2 McKelvey Lung Transplantation Center, Emory University School of Medicine, Atlanta, GA, USA; 3 Division of Pulmonary, Allergy and Critical Care Medicine, University of Pittsburgh, Pittsburgh, PA, USA; 4 Division of Pulmonary, Allergy and Critical Care Medicine, University of Louisville, Louisville, KY, USA; 5 Division of Pulmonary, Allergy and Critical Care Medicine, Atlanta VAMC, Decatur, GA, USA

**Keywords:** Lung Fibrosis, Thy-1, Fibrocytes, Extracellular Matrix, Fibronectin, TGF β1

## Abstract

Fibrotic lung diseases increase with age. Previously we determined that senescence increases tissue expression of fibronectin EDA (Fn-EDA) and decreases fibroblast expression of Thy-1, and that fibrocytes contribute to fibrosis following bleomycin-induced lung injury in mice. In this study we hypothesized that fibroblasts lacking Thy-1 expression produce an extracellular matrix that promotes fibrocyte retention and myofibroblast transdifferentiation, thereby promoting fibrogenesis. Young and old mice were treated with bleomycin intratracheally; fibrocytes in the bone marrow, blood, and lungs were quantified, and lung fibroblast Thy-1 expression assessed. Bone marrow-derived fibrocytes were cultured on matrices derived from Thy-1(+) or Thy-1(−) fibroblasts ± the pro-fibrotic cytokine TGFβ1. Older mice had more fibrocytes in their bone marrows at baseline and more fibrocytes in their lungs following bleomycin treatment. In parallel, lung fibroblasts in older mice had lower expression of Thy-1 at baseline that increased transiently 7 days after bleomycin treatment but then rapidly waned such that 14 days after bleomycin treatment Thy-1 expression was again markedly lower. Fibrocytes cultured on matrices derived from Thy-1(−) fibroblasts + TGFβ1 had increased gene expression for collagen type 1, fibronectin, Fn-EDA, and α-smooth muscle actin. In parallel, whereas the matrices derived from Thy-1(−) fibroblasts stimulated phosphorylation of Akt in cultured fibrocytes, the matrices derived from Thy-1(+) fibroblasts induced apoptosis. These findings suggest that senescence increases fibrocyte recruitment to the lung following injury and that loss of Thy-1 expression by lung fibroblasts promotes fibrocyte retention and myofibroblast trans-differentiation that renders the “aging lung” susceptible to fibrosis.

## 1. INTRODUCTION

Idiopathic Pulmonary Fibrosis (IPF) is a devastating progressive lung disease with an average survival of only 3 to 5 years [1]. The mechanisms underlying the initiation-nand progression of IPF are poorly understood and currently there are no effective treatments [2]. Aging or “senescence” is associated with increased susceptibility to injury and fibrosis [3–5]. However, there is little known about how senescence predisposes the lung to ineffective repair and increased fibrosis after injury.

Several factors have been linked to pathological lung fibrogenesis including circulating bone marrow-derived fibroblast progenitor cells, known as fibrocytes [6–8]. Fibrocytes are bone marrow-derived fibroblast progenitor cells characterized by their expression of both stromal markers (*i.e*. collagen types I and III and vimentin) and hematopoietic markers (*i.e*. CD45) [9]. Several studies, both in experimental animal model and humans, demonstrate that fibrocytes are recruited (via the CXCR4 axis) to sites of tissue injury including the lung where they contribute to pathological fibrosis [9,10]. Once recruited to injured tissues, fibrocytes may transdifferentiate into myofibroblasts and this speculation is supported by the fact that fibrocytes can be induced to differentiate into myofibroblasts by exposure to TGFβ1 *in vitro* [11]. However, the precise fate of fibrocytes in the lung has not been well elucidated, and it is unclear why fibrocytes promote pathological fibrosis in certain circumstances but not in others and why senescent lungs are more prone to develop fibrosis.

Under normal conditions within the lung, the extracellular matrix (ECM) is derived primarily from residential lung fibroblasts that are a heterogeneous population expressing different surface markers and displaying different functional characteristics. One of the most extensively studied surface markers of fibroblast heterogeneity is Thy-1 [12]. Several studies showed that Thy-1(+) fibroblasts differ from Thy-1(−) fibroblasts in many ways including the relative compositions of the ECM they produce [13,14]. Lack of Thy-1 expression is associated with increased TGFβ1 activation [15,16] and relatively greater fibrotic responses to bleomycin-induced lung injury in mice [12]. We previously determined that senescent lungs contain more fibroblasts lacking Thy-1 expression (both a decrease in the number of cells with Thy-1 expression and a reduction in Thy-1 expression per cell) and these lungs also express more TGFβ1 at baseline [17].

Accordingly, we hypothesized that lung senescence lung is associated with a progressive loss of lung fibroblast Thy-1 expression, and that these fibroblasts induce the production of a “pro-fibrotic” matrix that promotes fibrocyte trafficking and retention in response to injury where they undergo myofibroblast transdifferentiation and contribute to subsequent lung fibrosis. To test this hypothesis, we compared lung fibroblast Thy-1 expression and fibrocyte trafficking to the lung in young and old mice following bleomycin-induced lung injury.

## 2. MATERIALS AND METHODS

### 2.1. Animals

Young (3 month) and old (24 month) C57BL/6 mice were utilized. All studies were approved by the Institutional Animal Care and Use Committee (IACUC) at Emory University and conformed to institutional standards for the humane treatment of laboratory animals.

### 2.2. Bleomycin-Induced Lung Injury

Bleomycin-induced lung injury was performed as previously described [17]. Mice received either bleomycin (2.5 units/kg) in phosphate-buffered saline (PBS) or vehicle (PBS) alone intratracheally. Mice were euthanized at 7 and 14 days following injury; lungs, blood and bone marrow were harvested for analyses.

### 2.3. Lung Fibroblast Isolation, Characterization and Thy-1 Subpopulation Purification

Primary lung fibroblasts (PLFs) were harvested from all treatment groups as previously described [17]. PLFs (passage 1) were harvested and surface stained with anti-mouse Thy-1 conjugated with Phycoerythrin (PE) fluorescent and subjected to flow cytometry analysis for Thy-1 expression or sorting using Aria. Sorted cells were then cultured and expanded in fibroblast culture medium.

### 2.4. Analysis of Fibrocytes in Peripheral Blood, Bone Marrow and Lung

Cells from blood, bone marrow and lung were surface stained with PerCP-conjugated anti-mouse-CD45 and Phycoerythrin (PE)-conjugated anti-mouse CXCR4, per-meabilized and stained with a rabbit anti-collagen I antibody followed by FITC-conjugated goat anti-rabbit IgG secondary anti-body. Samples were analyzed on a FACS-Calibur and data were analyzed using the FlowJo soft-ware.

### 2.5. Fibrocytes Isolation and Characterization

**Figure 1:**
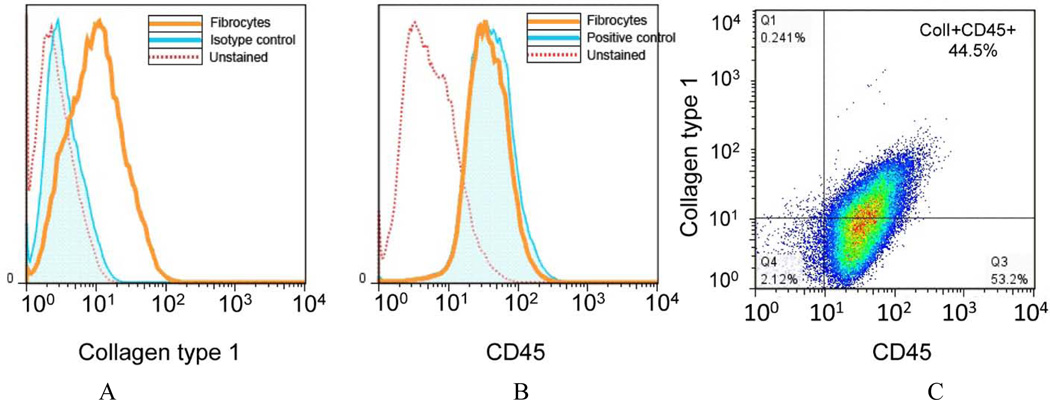
Fibrocyte isolation and purification. Fibrocytes were isolated from adherent cells cultured from total bone marrow. Cells were negatively selected for CD3e, CD14, CD19 and Ly6G and positively selected for CD45 by magnetic bead separation. Enriched fibrocytes were stained for CD45 and collagen type I for flow cytometry analysis. **(A)** shows a representative histogram of collagen type I staining in comparison to unstained and isotype control staining. **(B)** shows a representative histogram of CD45 staining in comparison to positive control (lymphocytes) and unstained cells. **(C)** shows a density plot of CD45 and Collagen type I staining, which show approximately 44.5% of cells are collagen type I+ and CD45+.

Total adherent bone marrow cells were obtained as previously described [18]. Cells were purified using the MAC system to eliminate CD3e, CD14, CD19 and Ly6G positive cells as well as CD45 negative cells. Purified cells were utilized for experiment or characterization (described below). For characterization, cells obtained from magnetic cell separation were stained with CD45 and Col-1 and analyzed by flow cytometry as described above (Figure 1). Our purity of the isolated (Col1 + CD45+) was approximately 44.5%.

### 2.6. Fibrocyte Differentiation on Fibroblast Subpopulation-Derived Matrix

Thy-1(+) or Thy-1(−) fibroblasts were cultured in medium containing 10% of fibronectin-depleted serum for 96 hours. Fibroblasts were lysed using sterile DOC lysis buffer [19] leaving insoluble assembled ECM on the culture surface. Following multiple washes with PBS, visualization with microscopy was performed to assess for any left over fibroblasts. Fibrocytes were cultured atop these surfaces. At 24 hours after initial plating, cells were incubated with 2 ng/mL of recombinant TGFβ1. Cells were harvested for analysis of phospho-Akt and total Akt expression (at 2 hours) and for mRNA expression (at 24 hours) and for α-SMA expression, and expression of cleaved caspase 3, TUNEL and Annexin V staining (at 72 hours).

### 2.7. Messenger RNA Expression Analysis

Messenger RNA from *in vitro* experiment was isolated, first-strand cDNA was synthesized as previously described [20]. Quantitative polymerase chain reaction (PCR) was performed with primers set for 18s, fibronectin (FN), FN EDA, collagen type I, and α-smooth muscle actin (α-SMA) and analyzed as previously described [17,20].

### 2.8. Protein Isolation and Analysis

Protein from cultured fibrocytes was harvested and Western Immunoblot was performed as previously described [17]. Blots were incubated with primary antibodies as follows; rabbit anti-mouse phospho-Akt, pan-Akt or cleaved caspase 3 at 1:1000, then followed by an appropriate secondary antibody and visualized via enzyme-linked chemiluminescence using the SuperSignal West Pico kit [17].

### 2.9. Immunofluorescent Staining for α-SMA

Cells were fixed and permeabilized and incubated with primary antibody; rabbit anti-mouse α-SMA at 1:200 at 4˚C overnight. Slides were incubated with a secondary antibody; goat anti-rabbit 488 at 1:500; DAPI nuclear stain was applied and visualized using the Olympus BX51. Cells were counted and the percentage of cells that stained positive for α-SMA was calculated from 5 random 20× magnification fields.

### 2.10. Analysis for Apoptosis by Terminal Deoxynucleotidyl Transferase dUTP Nick End Labeling (TUNEL) Assay and Annexin V Staining

TUNEL assay and Annexin V staining were performed using the *in situ* cell death detection kit, POD and Annexin V-FITC Fluorescence Microscopy Kit according to manufacturer’s protocol. Slides were visualized using the Olympus BX51. Cells were counted and the percentage of cells that stained positive for TUNEL or Annexin V was calculated from 5 random 20× magnification fields.

### 2.11. Statistical Analyses

One-way ANOVA was used for comparisons among groups, and post-test analysis using Bonferroni’s method performed if statistical significance was reached. Significant differences were accepted at a *P* level of <0.05.

## 3. RESULTS

### 3.1. Senescent Bone Marrow Contains Higher Number of Fibrocytes at Baseline and Senescent Lungs Retain more Fibrocytes Following Bleomycin-Induced Injury

**Figure 2:**
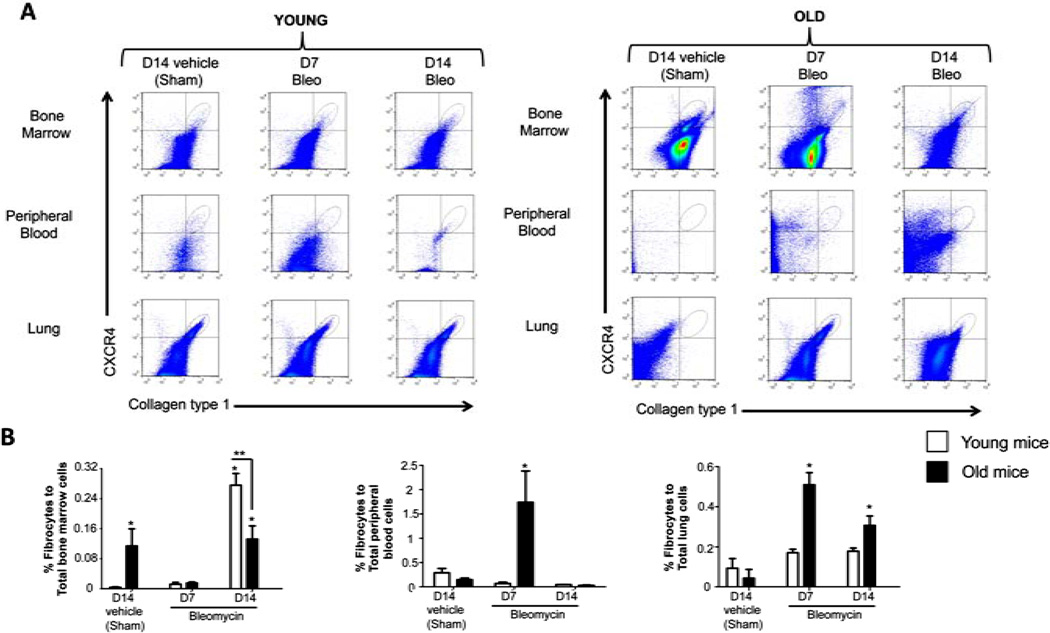
Fibrocyte trafficking into the peripheral blood and to the lungs is increased in old mice following bleomycin-induced lung injury. (A) shows representative scatter plots of CD45+ cells, gated for CXCR4 and collagen type I (fibrocytes) in the bone marrow, peripheral blood and the lung. (B) shows the percentages of fibrocytes in the bone marrow, peripheral blood, and the lungs of young mice (3 months old, open bars) and old mice (24 months old, closed bars) at 14 days following sham treatment with phosphate-buffered saline (PBS), and at 7 days and 14 days following bleomycin treatment. N = 5 - 6 in each group. ^*^P < 0.05 increased compared to young sham-treated group. ^**^P < 0.05 decreased compared to young bleomycin-treated mice at 14 days.

We first quantified the percentages of fibrocytes (CD45+, CXCR4+, Collagen type 1 + cells) in the bone marrow, peripheral blood and the lung in mice 14 days following sham treatment and compared these to the percentages of fibrocytes in these respective compartments in mice 7 and 14 days following bleomycin treatment. As shown in Figure 2 (Figure 2A shows representative flow cytometry histograms and Figure 2B shows the summary data), sham-treated old mice had a significantly higher percentage of fibrocytes in their bone marrow than sham-treated young mice; in contrast, there were no apparent differences in the percentage of fibrocytes in the peripheral blood or the lungs of sham-treated older or younger mice. By comparison, 7 days following bleomycin-induced lung injury there was a significant decrease in the percentage of fibrocytes in the bone marrows of old mice and a concomitant and significant increase in the percentage of fibrocytes in their peripheral blood and lungs, whereas there were no significant differences in any of these compartments in young mice 7 days following bleomycin-induced lung injury. In parallel, the percentage of fibrocytes in the lung remained elevated in old mice 14 days following bleomycin-induced lung injury, whereas there was still no increase in the percentage of fibrocytes in the lungs of young mice. Interestingly, young mice quite dramatically increased the percentage of fibrocytes in their bone marrow compartment 14 days following bleomycin-induced lung injury but not in their peripheral blood or lungs.

### 3.2. Bleomycin-Induced Lung Injury is Associated with an Increase in Thy-1 Expression by Lung Fibroblasts in both Young and Old Mice

**Figure 3:**
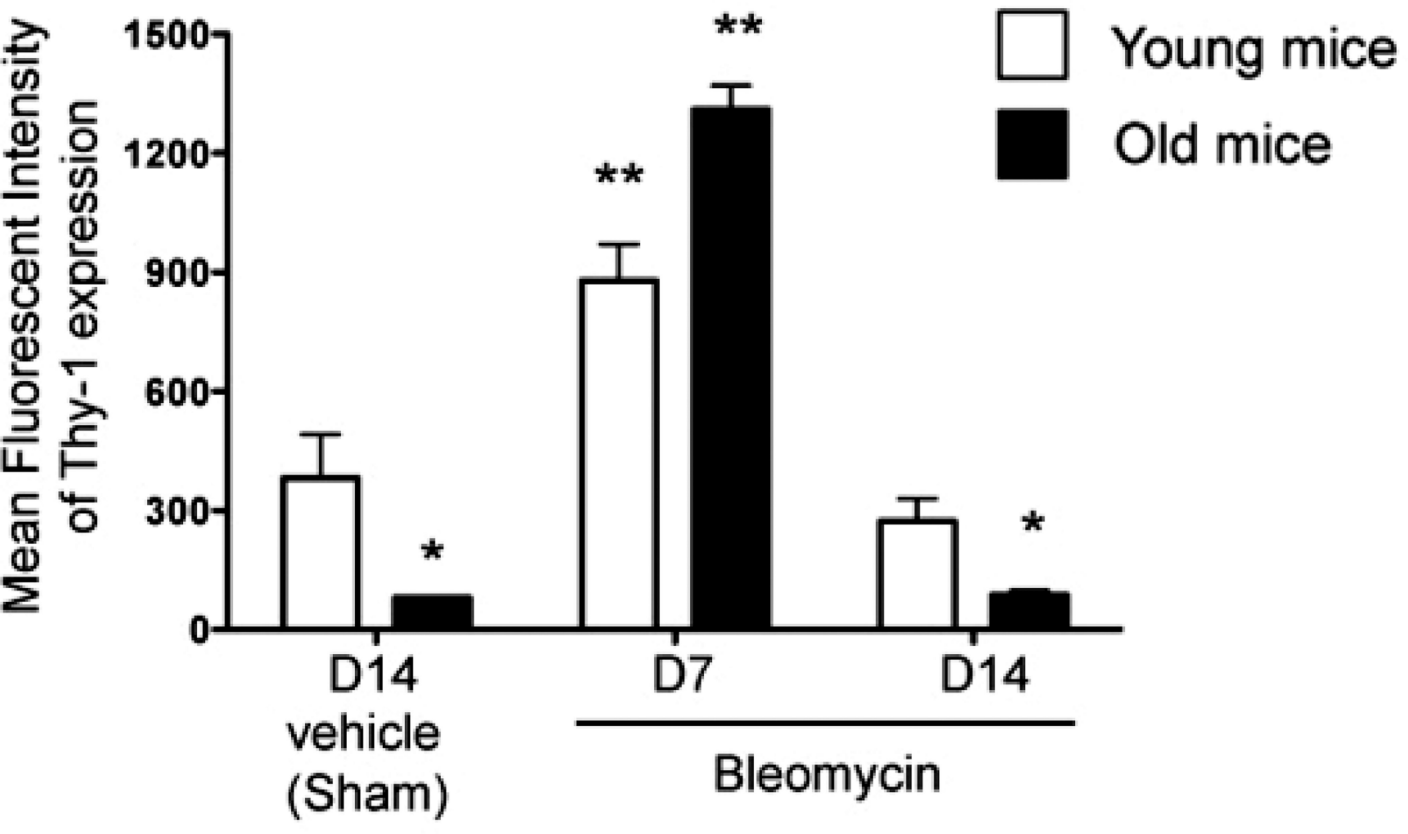
Lung fibroblasts from both young and old mice increase Thy-1 expression in response to bleomycin-induced lung injury. Primary lung fibroblasts were isolated from the lungs of young (3 months old, open bars) and old (24 months old, closed bars) mice at 14 days following sham treatment with phosphate-buffered saline (PBS), and at 7 days and 14 days following bleomycin treatment. Isolated fibroblasts were analyzed by flow cytometry for Thy-1 expression, as represented by mean fluorescence intensity (MFI). N = 3–6 mice per group. Data are represented as mean ± SEM. ^*^P < 0.05 decreased compared to young sham-treated group. ^**^P < 0.05 increased compared to young sham-treated group.

We previously showed that senescence is associated with a decrease in the expression of Thy-1 protein by lung fibroblasts in mice [17]. In parallel, it has been shown that inflammation alters endothelial cell Thy-1 expression [21]. We hypothesized that senescence could alter the response to injury by the lung fibroblast. Therefore, we next examined Thy-1 protein expression by lung fibroblasts isolated from young and old mice 14 days after sham treatment and compared this to Thy-1 protein expression by lung fibroblasts isolated from young and old mice 7 and 14 days following bleomycin-induced lung injury. As shown in Figure 3, fibroblasts derived from sham-treated old mice had significantly less expression of Thy-1 than fibroblasts from sham-treated young mice. Interestingly, lung fibroblast expression of Thy-1 increased significantly in both old and young mice 7 days following bleomycin-induced lung injury, but by 14 days had decreased back to levels seen in lung fibroblasts from sham-treated mice young and old mice, respectively.

### 3.3. The Extracellular Matrix (ECM) Derived from Thy-1(−) Fibroblasts Promotes Fibrocyte to Myofibroblast Transdifferentiation in the Presence of TGFβ1

**Figure 4:**
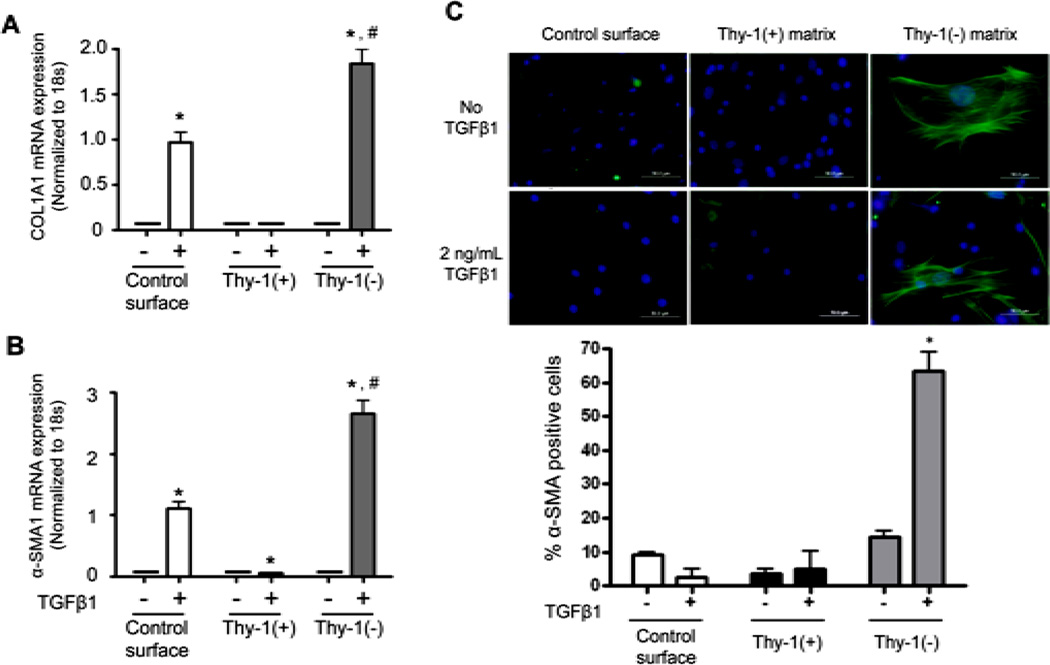
The extracellular matrix (ECM) derived from Thy-1(−) lung fibroblasts promotes fibrocyte-to-myofibroblast transdifferentiation in the presence of TGFβ1. Lung fibroblasts were isolated from young mice and sorted by FACS on a BD FACSAria cell sorter into Thy-1(+) and Thy-1(−) subpopulations. Samples with purity >90% were cultured in FN-depleted media. At 96 hours, cells were lysed to release endogenous extracellular matrix (ECM). Bone marrow-derived fibrocytes were cultured on a control surface (plastic) or on an ECM derived from either Thy-1(+) or Thy-1(−) fibroblasts in the presence or absence of TGFβ1 (2 ng/ml). At 24 hours after TGFβ1 treatment, cells were harvested for (**A**) collagen type I and (**B**) α-SMA mRNA expression as determined by real time RT-PCR. (**C**) At 72 hours, cells were harvested and immunostained for α-SMA protein expression and evaluated by confocal microscopy (upper inset). Original magnification 400x, scale bar = 50 µm. Summary **r**esults in the lower inset of panel C are the percentage of cells positive for α-SMA over 5 random 200× magnification fields per slide and are represented as mean ± SEM. ^*^P < 0.05 increased compared to fibrocyte control group without TGFβ1 treatment. ^#^*P* < 0.05 increased compared to fibrocyte control group with TGFβ1 treatment.

As shown in Figure 1, only the lungs of old mice had an increase in the percentage of fibrocytes following bleomycin-induced lung injury, and this increase was sustained for at least 14 days. This finding, coupled with the observation that lung fibroblasts from old mice expressed less Thy-1, led us to hypothesize that the extracellular matrix (ECM) derived from Thy-1(−) fibroblasts promotes fibrocyte recruitment and myofibroblast transdifferentiation. To test this hypothesis, we next compared markers of differentiation in fibrocytes cultured on the ECM produced by Thy-1(+) and Thy-1(−) fibroblasts in the presence or absence of recombinant TGFβ1. As shown in Figure 4A and **B**, there were no significant differences in Col1A1 or α-SMA mRNA expression between fibrocytes cultured on ECM derived from Thy-1(+) or Thy-1(−) fibroblasts in the absence of TGFβ1. In parallel, there were no significant differences in α-SMA protein expression between fibrocytes cultured on ECM derived from Thy-1(+) or Thy-1(−) fibroblasts in the absence of TGFβ1 (Figure 4C). However, in the presence of TGFβ1, fibrocytes cultured on ECM derived from Thy-1(−) lung fibroblasts significantly expressed their expression of Col1A1 and α-SMA mRNA (Figure 4A and B) and α-SMA protein expression (Figure 4C) as compared to fibrocytes cultured on ECM derived from Thy-1(+) lung fibroblasts. Interestingly, if anything the ECM derived from Thy-1(+) fibroblasts appeared to suppress fibrocyte α-SMA mRNA expression even in the presence of TGFβ1 (Figure 4B).

### 3.4. The Extracellular Matrix (ECM) Derived from Thy-1(−) Fibroblasts Stimulates Fibrocytes to Express Fibronectin-EDA in the Presence of TGFβ1

**Figure 5:**
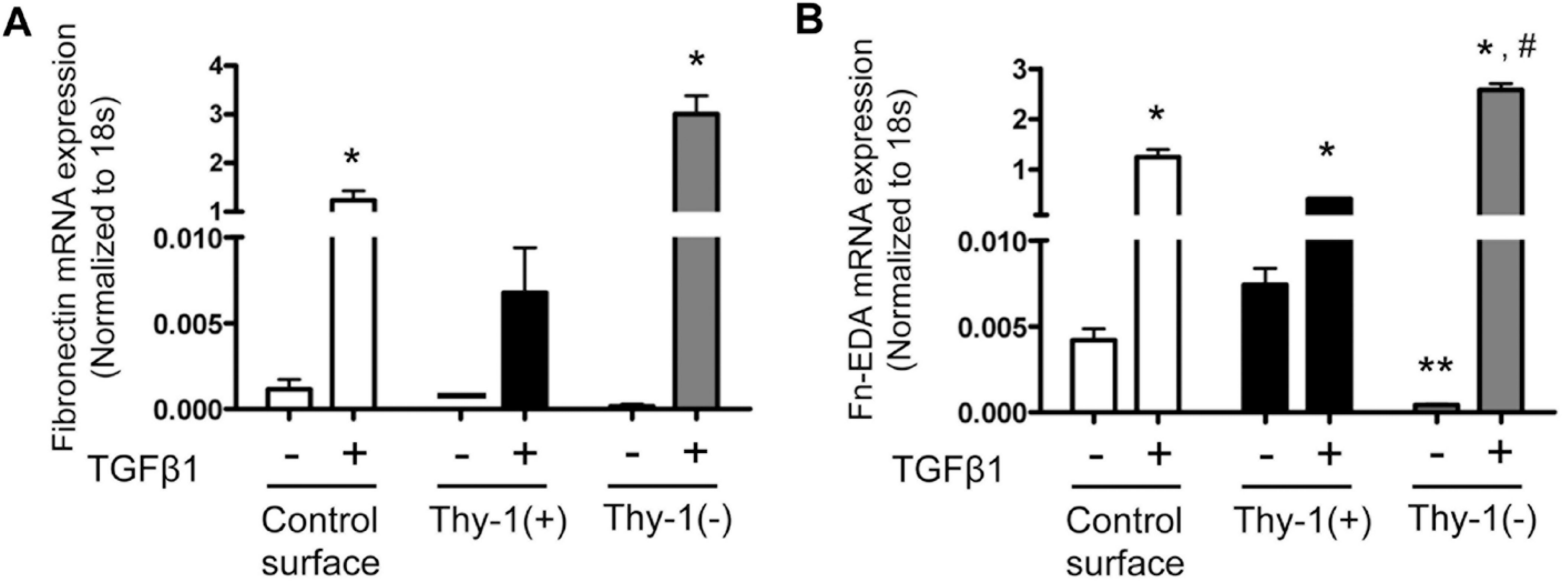
The extracellular matrix (ECM) derived from Thy-1(−) fibroblasts induces fibronectin-EDA gene expression in the presence of TGFβ1. At 24 hours following TGFβ1 treatment, cells were harvested and analyzed for (**A**) fibronectin (FN) and (**B**) fibronectin-E D A (FN-EDA) mRNA expression by real time RT-PCR. N = 3 mice per group. Data are represented as mean ± SEM. ^*^P < 0.05 increased compared to fibrocyte control group without TGFβ1 treatment. ^**^P < 0.05 decreased compared to fibrocyte control group without TGFβ1 treatment. ^#^*P* < 0.05 increased compared to fibrocyte control group with TGFβ1 treatment.

We previously showed that senescent lungs expressed higher levels of fibronectin-EDA mRNA at baseline and in response to bleomycin-induced injury [17]. We hypothesized that fibrocytes recruited to the lung in response to injury contribute to this increased fibronectin-EDA expression in senescent lungs via interactions with senescent ECM. To examine this, we compared fibronectin and fibronectin-EDA expression by fibrocytes cultured on ECM derived from Thy-1(+) or Thy-1(−) lung fibroblasts in the presence or absence of TGFβ1. As predicted, TGFβ1 stimulated fibronectin and fibronectin-EDA mRNA expression by fibrocytes in all groups (Figure 5). However, there was a significant increase in mRNA expression of both fibronectin (Figure 5A) and fibronectin-E DA (Figure 5B) when fibrocytes were cultured on an ECM derived from Thy-1(−) lung fibroblasts in the presence of TGFβ1. Interestingly and unexpectedly, in the absence of TGFβ1 the ECM derived from Thy-1(−) lung fibroblasts seemed to suppress fibrocytes fibronectin-EDA mRNA expression as compared to Thy-1(+) matrix without TGFβ1 group (Figure 5B), suggesting that the effects of TGFβ1 and Thy-1(−) fibroblast-de-rived ECM on fibrocyte expression of fibronectin-EDA are not simply additive but rather are synergistic.

### 3.5. The Extracellular Matrix (ECM) Derived from Thy-1(−) Lung Fibroblasts Stimulates Akt Phosphorylation, Which is Indicative of Cell Survival

**Figure 6:**
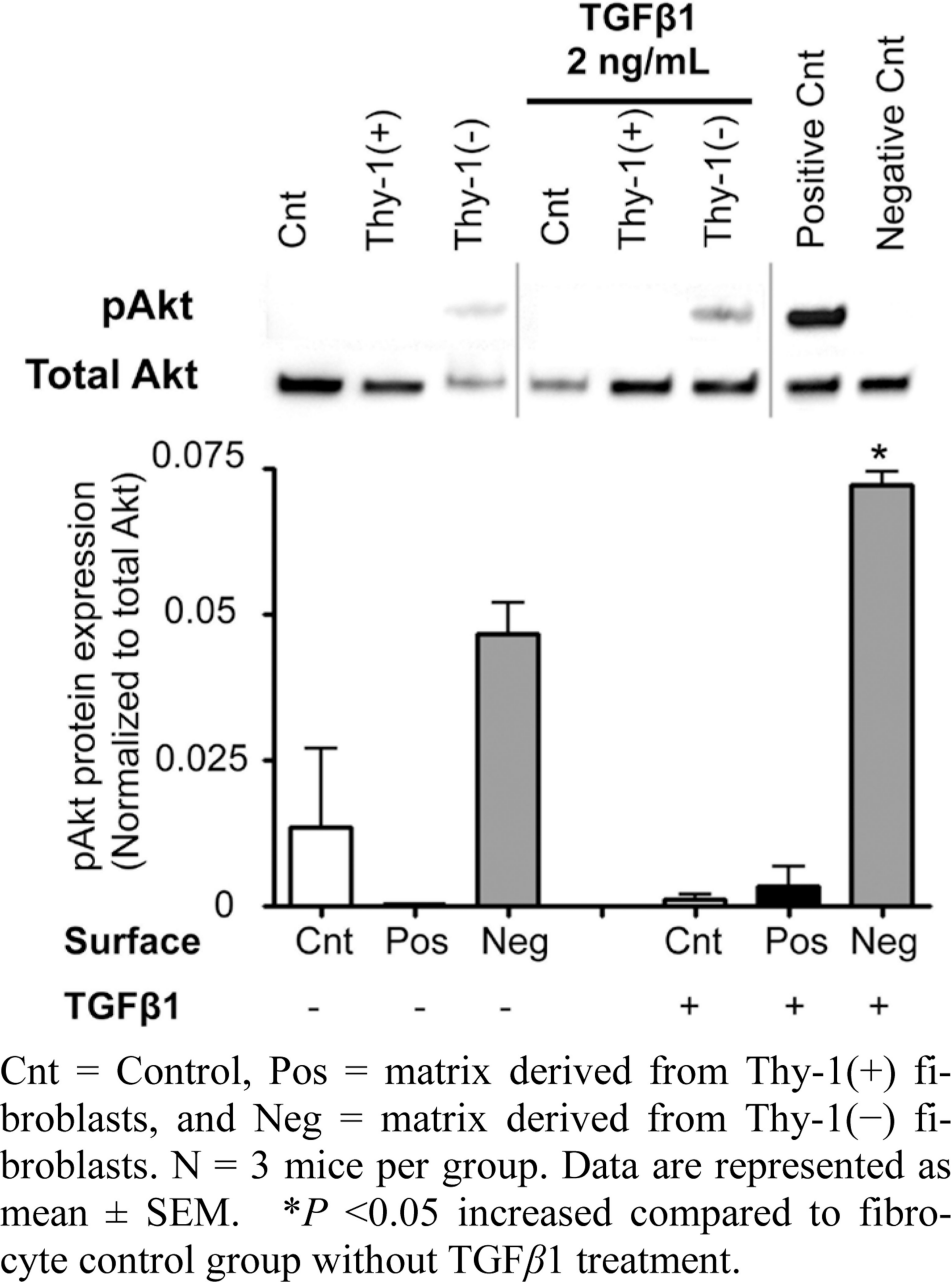
The extracellular matrix (ECM) derived from Thy-1(−) lung fibroblasts stimulates fibrocyte Akt phosphorylation. At 2 hours following TGFβ1 (2 ng/ml) treatment, cells were harvested for analysis of Akt phosphorylation by western blot. Positive and negative controls were cell extracts purchased from Cell Signaling. Upper inset shows representative western blots for total Akt and phospho-Akt expression. Lower inset shows densitometric analyses of phospho-Akt (pAkt) expression in relation to total Akt.

To examine how the ECM derived from Thy-1(+) and Thy-1(−) lung fibroblasts affects cell survival pathways in fibrocytes, we compared the expression of phospho-Akt and total Akt from fibrocytes cultured on these matrices. Some cells were stimulated with TGFβ1 (2 ng/ml) for 2 hours prior to harvesting for protein extraction and analysis. As shown in Figure 6, we determined that fibrocytes cultured on an ECM derived from Thy-1(−) lung fibroblasts had increased phosphorylation of Akt (pAkt) following TGFβ1 stimulation at 2 hours when compared to fibrocytes cultured on an ECM derived from Thy-1(+) lung fibroblasts, which in fact did not express pAkt even when stimulated with TGFβ1.

### 3.6. The Extracellular Matrix (ECM) Derived from Thy-1(+) Lung Fibroblasts Promotes Fibrocyte Apoptosis

**Figure 7:**
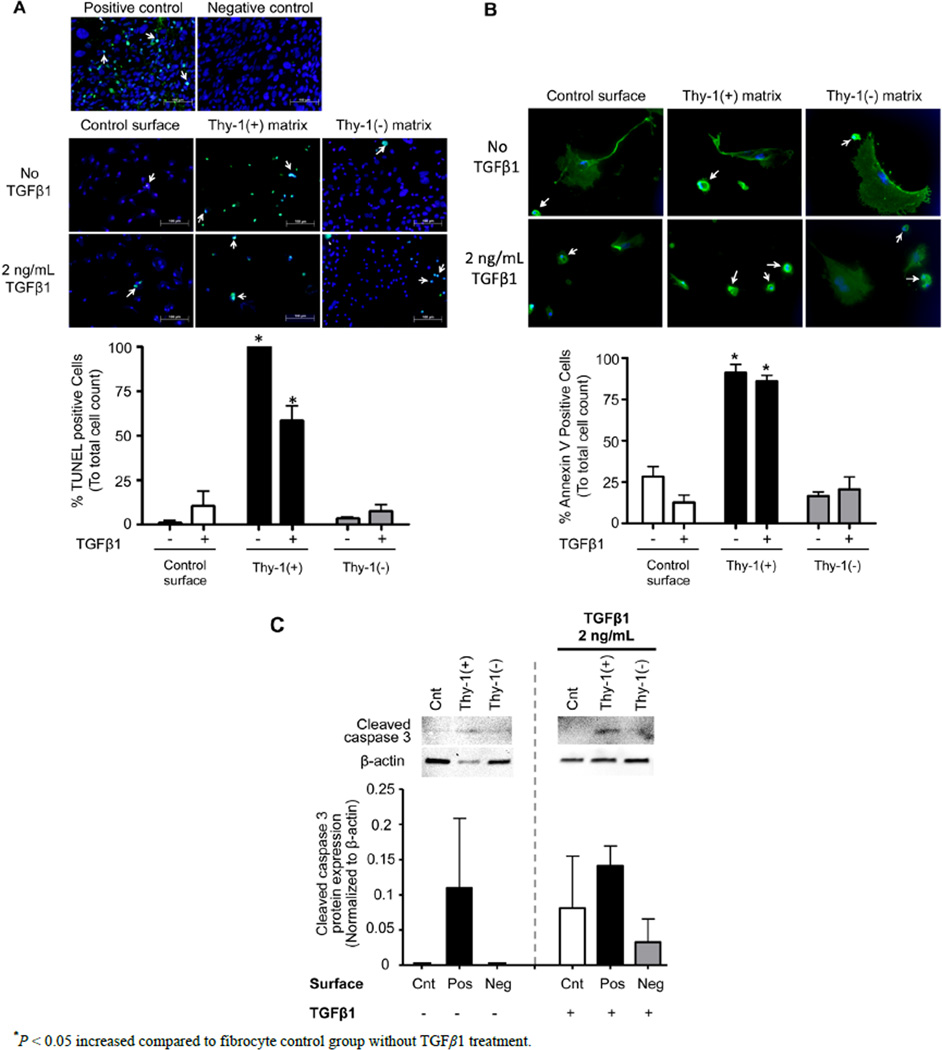
The extracellular matrix (ECM) derived from Thy-1(+) positive lung fibroblasts promotes apoptosis pathways in fibrocytes. Cultured fibrocytes were harvested and analyzed for markers of apoptosis by (**A**) TUNEL assay, (**B**) Annexin V expression, and (**C**) cleaved Caspase 3 protein expression 72 hours after TGFβ1 treatment. The upper inset in (**A**) shows representative microscopic images of the TUNEL assay(200×, bar = 100 µM). The lower inset in (**A**) is a bar graph representation of the percentages of TUNEL positive cells from 5 random 200× magnification fields per slide. The upper inset in (**B**) shows representative microscopic images of Annexin V staining (200×, bar = 100 µM) and the lower inset is a bar graph representation of the percentages of Annexin V positive cells analyzed from 6 random 200x magnification fields per slide. White arrows = cells positive for Annexin V. The upper inset in (**C**) shows Caspase 3 protein expression by western blot and the lower inset is a bar graph showing relative Caspase 3 protein expression by densitometric analyses. Cnt = Control, Pos = matrix derived from Thy-1(+) fibroblasts, and Neg = matrix derived from Thy-1(−) fibroblasts. N = 3 mice per group. Data are represented as mean ± SEM.

To examine the potential mechanisms by which the ECM derived from Thy-1(+) fibroblasts inhibits fibrocyte to myofibroblast transdifferentiation, we assessed fibrocyte apoptosis in response to culture on these matrices (by TUNEL assay and Annexin V). As shown in Figure 7, there was significant increase in TUNEL positive cells when fibrocytes were cultured on an ECM derived from Thy-1(+) lung fibroblasts, both in the presence and absence of TGFβ1, when compared to fibrocytes culture on an ECM derived from Thy-1(−) lung fibroblasts (Figure 7A), and these findings were supported by a similar increase in Annexin V staining (Figure 7B). As further evidence in support of these findings, we also determined that fibrocytes cultured on an ECM derived from Thy-1(+) lung fibroblasts showed a trend toward an increase in cleaved caspase 3 protein expression as compared to other conditions regardless of TGFβ1 treatment (Figure 7C).

## 4. DISCUSSION

In this study, we determined that senescent mice had more fibrocytes in their bone marrow, but comparable numbers of fibrocytes in the lung, when compared to young mice. However, following bleomycin-induced lung injury, senescent mice had more circulating fibrocytes and evidence for greater fibrocyte recruitment and/ or retention in the lung as compared to the younger mice, and this increase in the number of lung fibrocytes persisted up to 14 days after the injury. Consistent with our previous finding that lung fibroblasts isolated from senescent mice expressed less Thy-1 at baseline [17], we showed here that lung fibroblasts isolated from senescent mice expressed less Thy-1 at baseline and, despite a transient increase in lung fibroblast Thy-1 expression 7 days following bleomycin treatment, this expression rapidly waned to baseline low levels by 14 days following acute lung injury, whereas lung fibroblasts from younger mice maintained their higher levels of Thy-1 expression. In-parallel, we determined that isolated bone marrow-derived fibrocytes cultured *in vitro* on the extracellular matrix (ECM) derived from Thy-1 (−) lung fibroblasts, which comprise the majority of fibroblasts in the senescent mouse lung, expressed more collagen type I mRNA, and more α-SMA mRNA and protein, in the presence of TGFβ1 as compared to fibrocytes cultured on plastic or on ECM derived from Thy-1(+) lung fibroblasts. Further, we found that ECM derived from Thy-1(−) lung fibroblasts induced phosphorylation of Akt, which is part of a cell survival pathway, and promoted myofibroblast trans-differentiation, whereas the matrix derived from Thy-1(+) lung fibroblasts induced fibrocyte apoptosis in the presence of TGFβ1. Taken together, these studies provide novel evidence that Thy-1(−) fibroblasts, which represent the major lung fibroblast population in the lungs of old mice, produce a “pro-fibrotic” ECM that may promote fibrocyte retention and survival in the lung, as well as myofibroblast transdifferentiation, following injury and thereby render the senescent lung susceptible to fibrosis. These findings extend our previous studies in which we determined that senescent mice are more susceptible to lung fibrosis following bleomycin-induced injury [17].

Fibroblasts are normally present in the lung to help support the complex structure and function of the airways and their adjacent blood vessels. Following lung injury, the coordinated migration and proliferation of fibroblasts, coupled with the provisional ECM they produce, are essential steps for the development of granulation tissue, re-epithelization of injured airways, and the eventual removal of these activated fibroblasts [22]. Over the past decade, the precise origins of fibroblasts that have been implicated in fibrogenesis have been examined. These include the migration of residential lung fibroblasts, the transformation or “transition” of epithelial cells *in situ* (epithelial-mesenchymal transition or EMT), and recruitment and retention of a circulating population of fibroblast progenitor cells or fibrocytes [22–24]. Several reports over the past decade have suggested that fibrocytes play an important role in fibrogenesis of various tissues including the lung [7–9]. In clinical studies, fibrocytes have been shown to increase in numbers in the lungs of patients with pulmonary fibrosis, and the number of circulating fibrocytes was shown to correlate with the disease activity and mortality in these patients [8,24,25]. However, whether fibrocytes are simply a biomarker or in fact play a causal role in the pathogenesis of fibrotic lung diseases is unclear. In this study, we showed that senescent mice have more fibrocytes (as determined by cells with CD45+ CXCR4 + Col1+) in their bone marrow as compared to young mice. In parallel, even though they have the same number of fibrocytes in the circulation and in the lung at baseline, following bleomycin-induced lung injury senescent mice had significant increases in the percentages of both circulating fibrocytes and lung fibrocytes compared to young mice. These findings suggest that there are as yet undefined factors in the senescent lung that promote fibrocyte trafficking, retention, and/or survival in the lung following an acute injury. Based on our previous work as well as our findings in this new study, one intriguing candidate is the Thy-1(−) fibroblast that accumulates in the senescent lung.

It has been known that the microenvironment can influence cellular functions and behaviors [26–28]. For example, the stiffness or even contour of the culture surface can influence stem cell differentiation *in vitro* [29]. These microenvironments include other residential cells, soluble factors such as cytokines and chemokines, and the surrounding extracellular matrix [28]. Several factors have been shown to induce or promote lung fibrogenesis; these include TGFβ1, the EDA variant of fibronectin (Fn-EDA), and the relative shift toward a Thy-1(−) phenotype in lung fibroblasts [12,22,30]. We previously showed that the senescent lung microenvironment is different than that of the young lung, including the combination described above (increased TGFβ1 and Fn-EDA expression coupled with a decrease in fibroblast Thy-1 expression) [17]. We were particularly interested in Thy-1 expression because Thy-1(−) fibroblasts have been shown to be capable of activating TGFβ1 and Thy-1 null mice show increased fibrotic responses to bleomycin-induced lung injury as compared to wild type mice [12, 15]. These previous findings suggest that phenotypic changes in fibroblasts associated with the down-regulation of Thy-1 expression might promote fibroproliferation in the lung. This mechanism might be important in fibrotic lung diseases such as idiopathic pulmonary fibrosis (IPF) since affected individuals show accumulation of Thy-1 (−) fibroblasts in fibroblastic foci [31]. Previously, we identified a significant decrease in Thy-1 surface molecule expression by lung fibroblasts isolated from senescent mouse lungs [17].

It has been previously shown that lung fibroblasts increase their Thy-1 expression in response to inflammation [32,33]. We speculated that one of the mechanisms by which senescence increases fibrogenesis is by altering how the lung fibroblast responds to inflammation and injury. We were in fact somewhat surprised to find that there was in fact a comparable increase in Thy-1(+) lung fibroblasts following bleomycin-induced inflammation and injury in both young and old mice. These findings suggest that senescence may have not altered the initial lung fibroblast response to injury. However, we do not know whether there were any alterations in the function of these fibroblasts in the older mice, such as cytokine expression or ECM production, and by 14 days after acute lung injury this response had waned in the older mice. Clearly the transient increase (by recruitment and/ or phenotype switch) of a Thy-1(+) fibroblast subpopulation alone was insufficient to protect senescent lungs from an aberrant fibrotic response to bleomycin, and our results may reflect the importance of the baseline *(i.e*. “pre-injury”) ECM in effective lung repair following an acute injury. Specifically, the ECM derived from different subpopulations of lung fibroblasts appears to have a powerful influence on the behavior of bone marrow-derived fibrocytes that are recruited to the lung in response to an acute injury.

As described above, the microenvironment (including the ECM, resident cells, and their protein/receptor expression) influences cellular behavior. In this study we focused on the lung microenvironment and its interaction with recruited cells; specifically, fibrocytes and their possible role in aberrant repair and fibrotic response to injury in senescent lungs. We hypothesized that the age-related changes in the lung fibroblast phenotype alteration at baseline (particularly the loss of Thy-1 surface expression) is associated with the production of a “pro-fibrotic” matrix that in turn promotes the recruitment, retention, and transdifferentiation of fibrocytes, all leading to an aberrant injury response and the development of fibrosis. To assess this, we cultured fibrocytes on either a control culture surface (cell culture coated commercial available plastic ware) or on the ECM derived from Thy-1(+) or Thy-1(−) lung fibroblasts in the presence or absence of TGFβ1. Although the purity of fibrocyte we used in this particular study was approximately 44.5% and it was likely that the CD45+Col1- in the preparation were leukocytes, not mesenchymal stem cells; that could also differentiate into fibroblast or myofibroblast in the right condition, which could alter the interpretation of this study. We found that without TGFβ1 stimulation, there was no detectable difference in collagen type I and α-SMA mRNA expression or in α-SMA protein expression, which are the indicators of myofibroblast transdifferentiation. There was also no difference in fibronectin mRNA expression. Interestingly, Thy-1(−)-derived matrix appeared to suppress Fibronectin-EDA mRNA expression in the absence of TGFβ1 as compared to other culture conditions. However, in the presence of TGF 1β1, fibrocytes cultured on ECM derived from Thy-(−) lung fibroblasts expressed more collagen type I, fibronectin, fibronectin-EDA and α-SMA mRNA and more α-SMA protein compared to fibrocytes cultured on an ECM derived from Thy1(+) lung fibroblasts. Taken together, these data suggest that the ECM derived from Thy-1(−) somehow interacts with TGFβ1 to promote fibrocyte-to-myofibroblast transdifferentiation, whereas the ECM derived from Thy-1(+) fibroblasts does not. We further demonstrated that, in addition to the promoting fibrocyte-to-myofibroblast transdifferentiation in the presence of TGFβ1, the ECM derived from Thy-1(−) lung fibroblasts also promotes proliferation/survival pathways in the fibrocyte as reflected by increased phosphorylation of Akt, which is one of the components in the cell survival pathway [34], whereas the Thy-1(+)-derived ECM promoted fibrocyte apoptosis, as reflected by an increase in TUNNEL and Annexin V positive cells and trend toward an increase in expression of cleaved caspase 3. Whether or not this stimulation of the Akt pathway is through TGFβ1 receptors or through integrin receptor-ECM interaction, and whether or not this stimulation directly leads to fibrocyte to myofibroblast transdifferentiation, will need to be elucidated. Although to date we have not extensively characterized the ECM derived from Thy-1(+) lung fibroblasts cultured *in vitro*, it is plausible to infer that it is similar to the lung ECM in young mice and our experimental findings provide a potential mechanism to explain why young mice do not develop the degree lung fibrosis in response to bleomycin as do older mice.

In summary, we found that the senescent mouse lung recruits and retains more fibrocytes than the young mouse lung in response to bleomycin-induced lung injury. We speculate that this is in part due to the age-related change in lung fibroblast phenotype (*i.e*. loss of Thy-1 expression) and the pro-fibrotic extracellular matrix these senescent fibroblasts produce. Following lung injury, this aberrant matrix can influence both residential lung fibroblast behavior and the recruitment of fibrocytes that, under the additional influence of pro-fibrotic cytokines such as TGFβ1, can undergo myofibroblast transdifferentiation via activation of the Akt pathway and promote lung fibrosis. In contrast, the “healthy” extracellular matrix in the lung that is deposited earlier in life appears to be generated primarily by Thy-1(+) lung fibroblasts and actually promotes fibrocyte apoptosis, thereby limiting the fibrotic response following lung injury. Future studies will be necessary to further our understanding of the mechanisms by which fibrocytes interact with the extracellular matrix in the lung, including whether these interactions are through integrin receptors and/or cross talk between integrin receptors and TGFβ1 receptors. Alternatively, the lung ECM could influence the responses of fibrocytes via mechanotransduction mechanisms that depend on its relative structure and/or stiffness that may change with aging. Understanding these mechanisms will be critical in our quest to identify treatments that can slow or even arrest the progression of lung fibrosis.
